# Investigation Trp64Arg polymorphism of the beta 3-adrenergic receptor gene in nonobese women with polycystic ovarian syndrome

**DOI:** 10.18502/ijrm.v18i3.6712

**Published:** 2020-03-29

**Authors:** Farideh Zafari Zangeneh, Maryam Sarmast Shoushtari, Sahar Shojaee, Elahe Aboutorabi

**Affiliations:** ^1^Reproductive Health Research Center, Tehran University of Medical Sciences, Tehran, Iran.; ^2^Department of Chemical and Environment, Faculty of Engineering, UPM, Selangor, Malaysia.; ^3^Cell Therapy Center, Gandhi Hospital, Tehran, Iran.; ^4^Department of Genetics, Islamic Azad University Science and Research Branch, Tehran, Iran.

**Keywords:** Codon 64, Beta-3 adrenergic receptor, Polymorphism, Polycystic ovarian syndrome.

## Abstract

**Background:**

Polycystic ovary syndrome (PCOS) is a multifactorial and heterogeneous disease that has a potent inheritable component based on familial clustering. Despite many studies in the genetic field of PCOS, the genes that are involved in the causes of this syndrome have not been thoroughly investigated.

**Objective:**

The purpose of this study was to establish the occurrence of the Trp64Arg polymorphism of beta3 adrenergic receptor in non-obese women with PCOS.

**Materials and Methods:**

This cross-sectional study was performed on 100 women with PCOS and normal women as the control group in Imam Khomeini Hospital of Tehran in 2016-2017. Peripheral blood sample (2 cc) was obtained from two groups for genomic DNA based on the gene bank. Polymorphisms were genotyped by of using ADRB3 Trp64Arg. Then the DNA was extracted by genomic kiagen kit. The primer was analyzed for PCR based on gene bank by using Primer3 software and then confirmed by primer Blast tool at NCBI site to conformity to the beta-3 adrenergic receptor gene. The protein changes were assessment by the Clastal W software.

**Results:**

The sequence analysis presented in NCBI, transcript variant 1, with the code NM_000025.2, shows changes in the amino acid sequence of exon 1 in women with PCOS. Polymorphism in the codon 64 encoding the amino acid tryptophan (W) occurred in the nucleotide c.T190C, which changed the nucleotide T to C and then the amino acid sequence of the tryptophan was altered to arginine pW64R.

**Conclusion:**

T-C polymorphism is evident in the codon 64 of the adrenergic β3 receptor in patients with PCOS. Therefore, Beta3 adrenergic receptor gene polymorphism (Thr164Ile) associates with this syndrome in nonobese women.

## 1. Introduction

Polycystic ovary syndrome (PCOS) has heterogeneous clinical characteristics: polycystic ovarian morphology (PCOM), hormone imbalance, and metabolic disorders such as insulin resistance (IR), diabetes, and obesity. These conditions are the cause of infertility through ovarian and follicular maturation disorders (1, 2). Years of experimental and human studies show that the overactivity of the sympathetic nervous system (SNS) can be the main cause for signs and complications in PCOS. The adrenergic β${}_{3}$ receptor (ADR-B3) contains α1A, α1B, α1D, α2A, α2B, α2C, β1, and β2 subgroups. These adrenoceptors are located in the adipose tissue, vascular endothelium, and small intestine. The sympathetic activation of B3 is involved in cardio-inhibition, glucose uptake, lipolysis and relaxation of esophagus, bladder, and colon. The human β${}_{3}$ receptor gene has been localized to chromosome 8 (8p12-8p11.1). Β3ARs are also found in the brain, in areas such as the hypothalamus and the brain stem which can deliver multi-synaptic innervation to white and brain adipose depots (3). SNS-mediated brown adipose tissue (BAT) thermogenesis activity by diverse neurons found these areas in brain structures. SNS afferent neurons as thermoregulatory pathways control thermogenesis in BAT by thermoregulatory pathways with the interactions on the energy balance systems. The selective agonists of ADR-B3 can potently stimulate thermogenesis and lipolysis (4). Hadri and coworkers in 1997 showed that the transition from a fasted to a fed state in white and BAT in rats is associated with a decrease in β3-adrenergic receptor mRNA levels and β3-adrenergic receptor responsiveness. They suggested that there is a close relationship between β3-adrenoceptor expression, plasma insulin, and food intake (5). There are many reports of the association of the ADR-B3 Trp64Arg gene polymorphism with obesity and metabolic syndrome (6-8). The gene encoding ADR-B2 polymorphism has been reported in hypertension (9), asthma (10), and autoimmune disease (11).

In this study, we investigated the association of Trp64Arg (rs 4994) in the first cytoplasmic region (Uniprot accession p13945) as one variant in the ADRB3 gene in PCOS.

## 2. Materials and Methods

### Participants 

This cross-sectional study was performed in November 2015-2016 on women with PCOS. In tota, 100 women with PCO participated from the Reproductive Health Research Center of Imam Hospital. All women were aged 20-40 years and the body mass index (BMI) <28 kg/m${}^{2}$. They had no disease and no medication.PCOS diagnosis was according to the joint criteria of the European Society of Human Reproduction and Embryology and the American Society of Reproductive Medicine (ESHRE/ASRM) (12).

### Sampling 

In this study, 2cc of peripheral blood samples from the study (women with PCO) and control (normal) groups were collected in EDTA-treated tubes and froze at -20°C.

### DNA extraction

The extraction of genomic DNA from all samples was done by using of the QIAamp DNA BIA Mini Kit Qiamp (Cat ID // 51106). The genomic DNA concentration and purity were assessed by using of UNICO-spectrophotometer (S2100SUV). Purity was determined using the standard A260/A280 and A260/A230 ratios.

### Primer design

#### Primers used to amplify exon 1 from the ADRB3 gene

Primer design was performed using Primer3 software for NG_011936.1 (Homo sapiens adrenoceptor beta 3 (ADRB3), RefSeqGene on chromosome 8) considering exon 1 and 2 regions in CDS region and then by using primer blast tool at NCBI site, it was approved in accordance with the ADRB3 gene exon 1 and 2 in NM_000025.2 without any mismatch with another region of the human genome. The fragment of interest was amplified with the PCR primers listed in Table I.

#### Genotyping of adrenoceptor beta 3 gene: The amplification of sample for preparation of PCR

**Figure 1:**
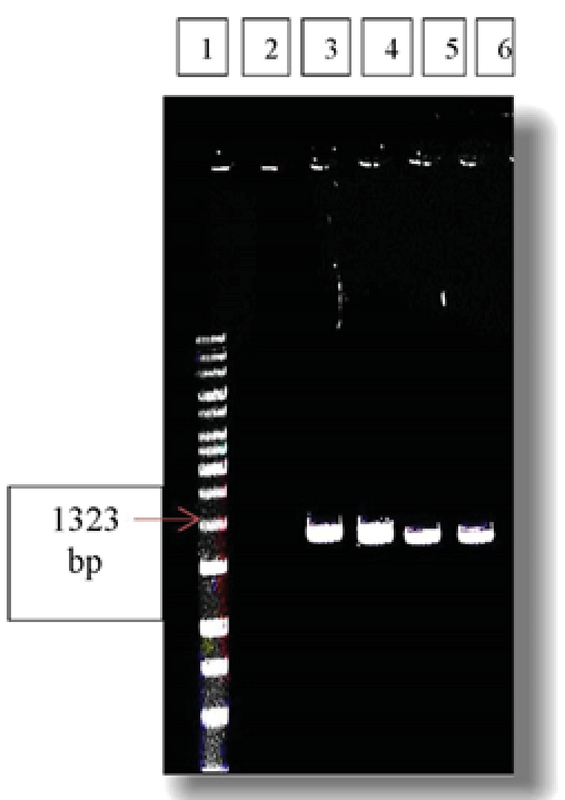
Representative agarose gel image showing amplification of exon 1 of ADRB3 gene by using its specific primers: (1) 100 bp DNA ladder; (2) Negative control of PCR; (3) Control sample 1; (4) Control sample 2; (5) Patient sample 3; and (6) Patient sample 4.

**Figure 2:**
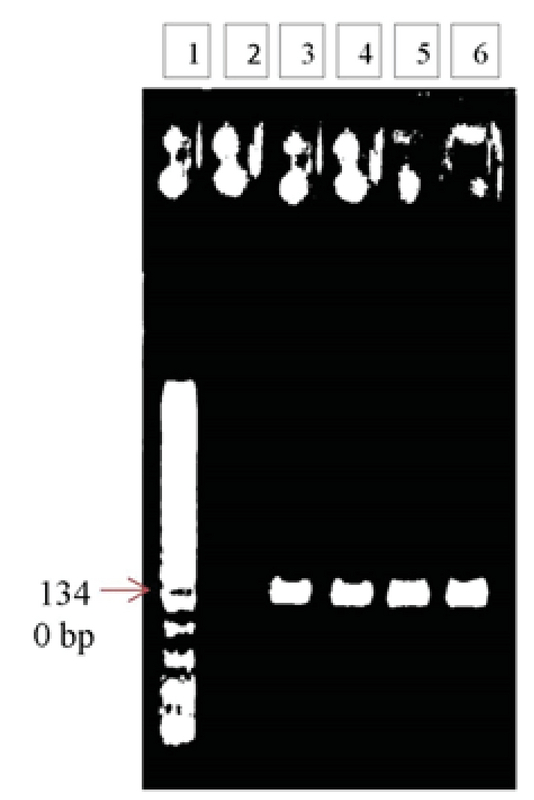
Representative agarose gel image showing amplification of exon 2 of ADRB3 gene by using its specific primers: (1) 100 bp DNA ladder; (2) Negative control of PCR; (3) Control sample 1; (4) Control sample 2; (5) Patient sample 3; (6) Patient sample 4.

25 *μ*L PCR mixture was prepared including: 20 ng of DNA, 10 pmol of each primer, 0.2 mmol/L of dNTPs, 2 mmol/L of MgCl${}_{2}$, and 1 U of Taq DNA polymerase. The thermal cycling situation for this study involved the original denaturation stage at 95°C for 10 min followed by 35 series of 95°C for 45 seconds, 59°C (exon 1) and 60°C (exon 2) for 1 minutes, and 72°C for 45 sec, and a final stage at 72°C for 10 minutes. All PCR products were exposed to electrophoresis in buffer of 1X TAE. The gel was marked with ethidium bromide (10mg/ml) and visualized under UV. The Gel documentation system was Transilluminator and photographed (Bio-Rad Laboratories) (Figures 1, and 2) and analysis after stored at 4ºC (table I). So, the fragments obtained were subjected to electrophoresis in 1% agarose gel, and the size of the fragments and quality of them were checked (Figures 1, and 2). All samples were subjected to sequencing for genotyping and patient sequence analysis. The results were reproducible without any discrepancies. The PCR results from four samples were shown on 1% agarose gel for exon 1 and 2, respectively, as shown below.

The sequence provided for ADRβ3 in NCBI, transcript variant 1, with the code NM_000025.2 was used for investigation of changes in amino acid.

**Table 1:** Primer used in amplification of ADRB3 gene


**Gene (NG_011936)**	**Primer sequence (F, R, 5' 3')**	**Product length (base pairs)**
**Exon 1**	F:TAGAGAAGATGGCCCAGGCT R:CGAGCCGTTGGCAAAGC	1323
**Exon 2**	F:GCTGGGTTGGAGTAGGGATG T:AGAGGTTGTGGAAAGGCTGG	1340

### Ethical consideration 

This study was approved by the Ethical Committee of Tehran University of Medical Sciences (code: IR.TUMS.VCR.REC.2016.1329). The study points were explained to these women before they participated in the study, and informed consent was obtained from all.

### Statistical analysis

IBM SPSS statistics (Statistical Package for the Social Sciences, version 24.0, SPSS Inc., and Chicago, IL) was used for analysis. To check the correlation between natural and mutated genotypes as well as homozygous and heterozygote genotypes Chi-square test (chi2) was used.. *P* < 0.05 was considered as statistically significant level.

## 3. Results

### PCR results

The quality and size of PCR products amplified for ADRB3 exon 1 and 2 sequences were analyzed using agarose gels. The comparison of PCR products with DNA size markers indicates that the PCR amplicons links to the expected PCR products. According to primer sequences (Table I), PCR product size of 1323 and 1340 was confirmed (Figures 1, and 2) and their quality were good for sequencing.

### Sequencing results

**Figure 3:**
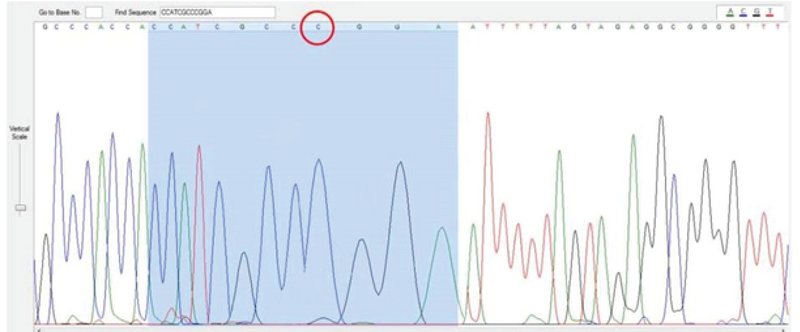
Sequencing of Codon64, c, mutant.

Our results show that codon 64 is associated with a polymorphism in women with PCOS. The point of mutation in PCR sequencing has been analyzed using Finch TV software in a woman with PCOS (Figure 3).

Our results show that there is a significant correlation between the presence of mutated genotype and PCOS. This result confirms that the mutagenic genotype can increases the chance of developing polycystic ovaries in women. OR: 2.546 (95% CI: 1.02-5.367) P: 0.012. The power of the study was also measured using the online GAS software (0.718), which was a significant measure that can indicate this study had high diagnostic power.

In this study, no significant correlation was found between homozygote and heterozygote genotype (p = 0.301), which is due to the high power of the study; it can be claimed that the sample size did not have an effect on statistical results. For complet, we used the Bootstrap test in which samples in each group were tested 1,000 times. Then, we used the Chi2 (it minimizes the sampling error) where these results had no difference (Table II).

**Table 2:** Comparison of mutation and zygote in Beta3 adrenoceptor gene between women with PCOS and control group


**Variables**	**Control**	**PCOS**	**Odd ratio (OR) (95%CI)**	**p-value**	**Statistical power**
	68 (85%)	69 (69%)		
**Wild mutant**	12 (12%)	31 (31%)	2.546 (95%CI:1.02-5.367)	0.012	
		68 (85%)			79 (21%)		
**Homozygote heterozygote**	12 (15%)	21 (21%)	1.506 (95%CI: 0.691-3.286)	0.301	0.718
	Data presented as n (%); Chi-square					

## 4. Discussion

In the present study, a significant association of Trp64Arg beta 3-adrenoceptor gene polymorphisms was observed in non-obese women with PCOS (BMI < 28 kg/m${}^{2}$). Our result suggests that women with this mutation genotype have a significantly increased risk of PCOS (OR = 2.546, 95% CI: 1.02-5.367).

ADR-β agonists are potent activators of BAT in mice and humans (13). All sub-groups of Adrenoceptors β (β${}_{1-3}$) have an active role in the metabolic processes like glycogenolysis, lipolysis, and insulin secretion. These adrenoceptors can cause metabolic syndrome (Mets) and PCOS. Familial and twin studies show that there is a genetic factor in women with PCOS with a polygenic pattern of heredity (14, 15). The studies on the PCOS exposure genes show that mostly these genes are associated with insulin sensitivity (16), sex hormone regulation (17), and metabolic disorders (18).

Energy metabolism can be a reliable predictor of weight gain. Overweight and obesity increase the incidence of PCOS and so the increasing of adiposity is considered a dominant characteristic in 40 to 60% of women with PCOS (19). There are at least four metabolic parameters that can be predictive of weight gain: low metabolic rate, low fat oxidation rates, low spontaneous physical activity, and low activity of SNS.

The BAT, as a specific kind of adipose tissue with high activity, plays an important role in the metabolism of the body due to its high mitochondrial levels (20). Females with intact ovaries who are more insulin sensitive are protected from metabolic disorders because their BAT ratio is higher than the total fat (21-23). In an adult, Wang and coworkers showed that BAT is strongly activated by an increase in the circulating catecholamine concentration and there is a significant amount of BAT expressing β3-adrenergic receptor (23). Recent studies show that there is an important role of the ADRβ3 axis in human energy metabolism. This finding is confirmed by a common genetic codon of Trp64 → Arg64 in ADRβ3, which can lead to metabolic abnormalities such as low levels of energy expenditure, high BMI (24), central obesity (25), hyperinsulinemia/IR (26), high blood pressure (BP) (27), type 2 diabetes (28), and even with gestational diabetes mellitus (29). These problems are also present in the symptoms and complications of women with PCO.

Studies of the last decade show that β3-AR controls BAT thermogenesis in humans (30, 31). The SNS as the main regulator of BAT can be firing the SAS sub serving BAT for releasing of noradrenaline (NA) and activation of β3 subtype (β3-AR). This trigger activates the lipolysis process and mitochondrial uncoupling in BAT (31). A study on the BAT as a thermogenic machine, as well as the SNS-BAT axis, can help us to understand the complex etiopathology of Mets and PCOS.

ADR-B${}_{3}$ (8p11.23) is essentially expressed in white/brown adipose tissue and is involved in thermogenesis thermogenesis and in the vascular endothelium for glucose uptake, lipolysis and cardio-inhibition/relaxation. The hypermethylation of this gene in visceral tissue leads to metabolic disorders (32).

In the present study, we investigated the Trp64Arg polymorphism of the beta 3-adrenergic receptor gene. For the selection of this codon, we must consider two important principles in women with PCOS as a dynamic study of two interwoven aspects: (1) epigenetic mechanisms and (2) neuroendocrine (autonomic/HPA/CRH imbalance) disorder.

Novel aetiopathological and treatment concepts can be raised from the fact that hypothalamic-pituitary-adrenal (HPA) axis dysfunction in PCOS like Mets could be initiated from sympathetic hyper activity in two syndromes. The homeostatic regulation of food intake is controlled by the HPA axis through many cross-links in the neural pathways of the neuroendocrine system (33). Firstly, it is the neurons of corticotrophin-releasing hormone (CRH) that contains the initial component of the HPA axis and are located in the paraventricular nucleus (PVN) of the hypothalamus, which is a major center for controlling the feeding behavior (34). One of the other affective factors in BAT thermogenesis is the CRH, which is another important regulator of energy storage and adaptive thermogenesis. CRH acts via specific G-protein-coupled receptors. Real time RT-PCR and immunofluorescence confirmed that CRH (CRH-R1/ R2) can target in skeletal muscle and two types of adipose tissue: white adipose tissue (WAT) and BAT (35). CRH-R1/R2 receptors have been found in the stromal cells, thecal and follicular fluid in human ovaries. Ovarian CRH-like NA regulates steroidogenesis, follicular maturation, ovulation, and luteolysis (the so-called “aseptic” inflammatory) processes. In human, peripheral infusion of CRH stimulates fat oxidation and thermogenesis without changing lipolysis in adipose tissue or sympatho-adrenal activation. In normal female ovaries, there is no CRH in the oocyte of primordial follicle (36). But in the theca cells of womwn with PCO, CRH is associated with the rich of sympathetic nerves (37). In women with PCOS, the presence of CRH with its inhibitory role in oocyte maturation might be accompanied with the occurrence of follicle atresia that is observed in this syndrome (38). Zangeneh and coworkers in 2017 showed that hyponeurotrophinemia and decreasing CRH level in the serum of nonobese women with PCOS could reflect a deficiency of neuronal stress adaptation (39). This finding shows that central CRH and ovary CRH in women with PCOS is different from normal. Solinas and coworkers in 2006 for the first time reported the skeletal muscle's thermogenesis directly stimulates by CRH. They demonstrated that this thermogenic effect requires both phosphatidylinositol 3-kinase and AMP-activated protein kinase signaling, which can control the thermogenesis in this tissue by its protection against skeletal muscle lipotoxicity and IR (40). IR in women with PCO is not generalized in all tissues. In rat modeling of the knock-out of insulin receptors, hyperandrogenism and anovulation enhanced (41). Defects in metabolic function by insulin can occur in adipose and muscle tissues, but in the ovaries, mitogen and steroidogenic actions are maintained (42). Most women with PCOS have metabolic IR, partly due to obesity or due to the genetic predisposition. Ovarian insulin receptor can increases the response of theca cells to LH, and by increasing the expression of P450c17 and 3β-HSD2 leads to elevate of androgen production (43). In women with PCO, IR as a metabolic abnormality has a significant link with chronic inflammation, and polycystic ovarian syndrome is a low-level chronic inflammation disease. Many studies show that this inflammation has an essential role in the IR and metabolic consequences in women with PCOS (44, 45). The set point of body weight is the balance between energy intake and energy expenditure, and the central role of insulin axis in energy imbalance can contribute to the pathogenesis of obesity. Insulin as the mediator of feeding-related can increase thermogenesis. Metabolic activity of BAT can be activated by agonists of β3-adrenoceptor, and NA transporter (NAT) can block it which is measurable using [18F] fluorodeoxyglucose positron emission tomography/computed tomography (PET/CT) in rat. By this method, Baranwal and coworkers showed that the beta-3-adrenergic agonist CL316, 243 has a potential role on BAT for modulating blood glucose levels (46).

Studies show that pharmacogenetics of β3AD agonists and antagonists of NAT can help us to treat obesity and diabetes (47, 48).

All of these bodies of evidences more confirm that PCOS is a heterogeneous disease with metabolic disorders and on the base of our hypothesis; Trp64Arg polymorphism of beta3 adrenoceptor can increase the chance of PCOS.

## 5. Conclusion

The genomic studies help us for novel aetiopathological and treatment concepts of PCOS. T-C polymorphism is evident in the codon 64 of the ADR-B3 in patients with PCOS and Beta3 gene polymorphism (Thr164Ile) associated with this syndrome. We need to study the future research that investigated the interactions of risk genotypes, environmental factors, and epigenetic encoding in the pathophysiology of PCOS.

##  Conflict of Interest 

The authors declare that there is no conflict of interest.
